# A New Acoustic-Based Approach for Assessing Induced Adulteration in Bovine Milk

**DOI:** 10.3390/s21062101

**Published:** 2021-03-17

**Authors:** Marcos Messias dos Santos Junior, Bruno Albuquerque de Castro, Jorge Alfredo Ardila-Rey, Fernando de Souza Campos, Maria Izabel Merino de Medeiros, José Alfredo Covolan Ulson

**Affiliations:** 1 Department of Electrical Engineering, Sao Paulo State University, Bauru 17033-360, Brazil; marcos.messias@unesp.br (M.M.d.S.J.); fernando.campos@unesp.br (F.d.S.C.); alfredo.ulson@unesp.br (J.A.C.U.); 2 Department of Electrical Engineering, Universidad Técnica Federico Santa María, Santiago de Chile 8940000, Chile; jorge.ardila@usm.cl; 3 Sao Paulo State Agrobusiness Agency, Bauru 17034-285, Brazil; medeiros@apta.sp.gov

**Keywords:** bovine milk adulteration, instrumentation systems, piezoelectric transducers, signal processing analysis

## Abstract

Milk is an important dietary requirement for many populations due to its high nutritional value. However, increased demand has also made it prone to fraudulent activity. In this sense, scientists have sought to develop simple, low-cost, and portable techniques to achieve quality control of milk in industry and farms as well. This work proposes a new instrumentation system based on acoustic propagation and advanced signal processing techniques to identify milk adulteration by industrial contaminants. A pair of transmitter-receiver low-cost piezoelectric transducers, configured in a pitch-catch mode, propagated acoustic waves in the bovine milk samples contaminated with 0.5% of sodium bicarbonate, urea, and hydrogen peroxide. Signal processing approaches such as chromatic technique and statistical indexes like the correlation coefficient, Euclidian norm and cross-correlation square difference were applied to identify the contaminants. According to the presented results, CCSD and RMSD metrics presented more effectiveness to perform the identification of milk contaminants. However, CCSD was 2.28 × 10^5^ more sensitivity to distinguish adulteration in relation to RMSD. For chromatic clustering technique, the major selectivity was observed between the contamination performed by sodium bicarbonate and urea. Therefore, results indicate that the proposed approach can be an effective and quick alternative to assess the milk condition and classify its contaminants.

## 1. Introduction

Nowadays, industry and science have grown attention to develop sensors aimed to guarantee the quality control in the food industry, avoiding several types of food adulteration. In this context, due to its high nutritional value, bovine milk is an important component of human nutrition and is used in several processes in the food industry [1,2,3,4,5,6,7]. However, the increased demand has also made it prone to fraudulent activity, and numerous adulteration cases have been reported in the last decade. Milk adulteration can occur by incorrect handling. Nevertheless, when done purposely, the main goal of milk adulteration is to extend its shelf life and obtain profits by increasing its volume.

Hydrogen peroxide and sodium bicarbonate are two substances used as a milk contaminant aiming to extend shelf life. These adulterants are commonly added to slow down the microorganism’s growth process. Another harmful substance is urea, which is added to maintain the density of the milk [1,2,3,4,5,6,7]. Apart from the economical profits, milk adulteration is an important issue since the adulterants can lead to several types of human health effects such as vomiting, diarrhea and abdominal pain. For example, contamination with hydrogen peroxide damages the stomach cells, which can lead to gastritis and inflammation of the intestine [1]. Urea can cause irregular heartbeat and muscle cramps [1]. Therefore, this is a critical concern to product consumers and the industry.

In order to verify the degree of adulteration to guaranty health safety, numerous approaches such as: Fourier transform infrared (FTIR) spectrometry [8], high-performance liquid chromatography (HPLC) [9], electrical impedance [6], capillary electrophoresis (CE) [10], optical sensors [11,12], scanners, freezing point instruments, etc., have been proposed for milk inspection. Also, parameters such as density, pH, microbial inhibitors, etc. can be analyzed by chemical methods. The objective is verifying the degree of contamination to ensure health safety.

Although many studies have reported the effectiveness of these adulteration detection methodologies, many practical issues have limited their practical use in industry and farms. Usually, these solutions take time, tend to be expensive or require a specific laboratory environment. Therefore, the development of low cost, portable and automated methodologies to identify milk adulteration is crucial to the improvement of the quality control in the food industry and farms.

As explained, the fraudulent activity on milk is characterized by the addition of contaminants which may alter the nutritional properties of this valuable food. One of the most promising non-invasive techniques to determine information about liquid content is the acoustic characterization (AC) test. Typically, the material properties are related to the acoustic parameters which allow material characterization with the wave propagation proprieties [13]. 

This work proposes a new instrumentation system able to identify and classify milk adulteration by industrial contaminants like bicarbonate, urea, and hydrogen peroxide. Applying a pair of transmitter-receiver piezoelectric transducers, configured in a pitch-catch mode, acoustic waves were propagated in the bovine milk samples. Aiming to assess the milk condition, advanced signal processing analysis was applied. Metrics such as root mean square deviation (RMSD), correlation coefficient deviation metric (CCDM), and cross-correlation square difference (CCSD) [14,15,16], were used to differentiate the acoustic signals from samples of raw and adulterated milk. As opposed to traditional works like [17] the proposed methodology does not rely on the calculus of velocity and attenuation parameters of acoustic waves. As an important advantage, the proposed statistical methodology can allow implementing a smart sensors network aiming to provide automated quality control to the industry/agriculture 4.0. In addition to the development of a methodology to assess the milk condition, new results proved that the chromatic technique can be used to identify, concomitantly, several types of adulterants, as opposed to the work presented in [18], which select just one adulterant.

This article is divided into five sections. In Section 2 the new instrumentation system is presented, followed by the experimental setup in Section 3. The results and discussions are shown in Section 4 and, the conclusion of the article is presented in Section 5.

## 2. New Instrumentation System to Assess Bovine Milk Adulteration

**Figure 1:**
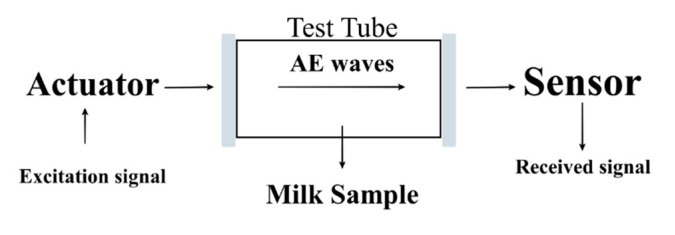
Acoustic liquid characterization to assess adulteration in bovine milk.

As previously presented, this work proposes a new acoustic-based instrumentation system, which is capable to identify and classify milk adulteration by industrial contaminants like bicarbonate, urea, and hydrogen peroxide. The theoretical background of acoustic propagation and signal processing analysis used in this article is presented in this section. Basically, the proposed work consists of an acoustic characterization system in which a signal is sent through milk contaminated or not contaminated and then received after its transmission (Figure 1). Statistical indexes applied to the received signals were used to determine whether the samples of the milk are contaminated or not. The chromatic technique was used to determine the type of milk contaminant.

### 2.1. Acoustic Liquid Characterization

In this work, a pair of transmitter-receiver piezoelectric transducers, configured in a pitch-catch mode, was used to perform the characterization of adulterated bovine milk by propagating acoustic waves in samples of milk. 

Figure 1 shows an example of a test tube which a piezoelectric transducer is configured as an actuator and another set as a receiver. Acoustic waves can be propagated in the milk samples by the excitation of the actuator, and the sensing signal will be related to the acoustic properties of the material sample. According to the acoustic theory, the transmission coefficient T can characterize the material capability to propagate acoustic waves and it is defined as [13]:

(1) $$T\;=\;\frac{1}{{\left(1+\frac{\beta \alpha L}{2}\right)}^{2}+\left(\frac{\beta^{2}-4}{4}\sin^{2}\left(\frac{2\pi fL}{c}\right)\right)}$$

where α is the wave attenuation coefficient, *L* is the length of the test tube, *f* is the frequency of the emitted wave, *c* is the wave speed. *β* is defined as a relationship between the acoustic impedance of the test tube wall (*Z_w_*) and the acoustic impedance of the liquid sample (*Z_l_*):

(2) $$\beta \;=\;\frac{Z_{w}}{Z_{l}}$$

The parameters α, *c* and *Z_l_* depend on the material that will be analyzed, *Z_w_* and *L* are related to the geometry and properties of the test tube, which are constant, and *f* is according to excitation signal applied.

By observing the parameters of Equations (1) and (2) it is clear acoustics can be a powerful tool to distinguish liquid materials. Considering the same excitation signal for all cases, the pattern of the wave propagation can be altered according to the adulterants added in the milk, which produces differences in the received signal by the sensor. Thus, statistical metrics that allow the comparison between the signals from samples of pure and contaminated milk can be an important tool to assess the milk condition. The next section will present the signal processing parameters used in this novel methodology. 

### 2.2. Statistical Index Approach

Among several signal comparison metrics, CCDM, RMSD and CCSD are effective for diagnosing differences in signals in time and frequency domain [14,15,16]. In this scenario, by applying acoustic waves in a sample of raw and adulterated milk, this work proposes the application of CCDM, RMSD and CCSD to distinguish pure from contaminated milk samples. 

To calculate these metrics, two types of received signals were considered, after the actuator excited the test tube with the acoustic waves: *S_r_*(*t*) and *S_a_*(*t*). The first, taken as reference (*S_r_(t)*), is extracted when the milk sample is considered pure. The second (*S_a_(t)*) is extracted after suspected contamination or sequentially in the case of the online monitoring systems. As discussed in the previous sub-section, the raw and adulterated milk may present different wave propagation patterns. Therefore, the difference between *S_r_*(*t*) and *S_a_*(*t*) may be an indicative of contamination. The calculation CCDM, RMSD and CCSD can quantify differences between two signals.

The RMSD, based on the Euclidean norm and the CCDM, based on the correlation coefficient [15], are defined as:

(3) $$RMSD\;=\;\overset{tf}{\underset{t=ti}{\sum }}\sqrt{\frac{{\left[\left(s_{a}\left(t\right)\right)-\left(s_{r}\left(t\right)\right)\right]}^{2}}{{\left(s_{r}\left(t\right)\right)}^{2}}}$$

(4) $$CCDM\;=\;1-\frac{\mathrm{cov}[\left(s_{a}\left(t\right)\right)-\left(s_{r}\left(t\right)\right)]}{\sigma_{r}\sigma_{a}}$$

where *S_r_*(*t*) is the voltage of the received signal taken from pure sample inspection, and *S_a_*(*t*) is the voltage of the received signal taken from a contaminated milk sample. *α_a_* and *α_r_* correspond to the standard deviation of the contaminated and raw milk sample, respectively. “cov” is the covariance of *S_r_*(*t*) and *S_a_*(*t*). *tf* and *ti* are, respectively, the initial and final time instant of the analyzed signal. In this article, the full time of the signal was applied to calculate the indexes.

The CCSD index is based on the cross-correlation function given by [14,19]:

(5) $$CCSD\;=\;\sum^{\text{​}}{\left(R_{s_{a}\left(t\right),s_{r1}\left(t\right)}-R_{s_{r1}\left(t\right),s_{r2}\left(t\right)}\right)}^{2}$$

where *R_sa_*_(*t*)*, Sr*1(*t*)_ is the cross correlation produced by a measurement *S_a_*(*t*) (a voltage signal extracted from a milk sample that we need to know whether are contaminated) and the signature *S_r_*_1_(*t*) obtained from pure milk. *R_sr_*_1(*t*)*, Sr*2(*t*)_ is the cross correlation produced by two signals *S_r_*_1_(*t*) e *S_r_*_2_(*t*), extracted from pure milk samples and used as reference of the system.

According to results presented in the following sections, RMSD and CCSD were able to differentiate pure milk from contaminated milk.

### 2.3. Chromatic Clustering Technique 

The chromatic technique (CT) stands out to be an effective approach of pattern recognition or data grouping, allowing the analytical extraction of information from a complex set of signals whose temporal and spectral characteristics cannot be easily identified [20,21,22]. In this context, CT was applied to the signals captured by the receiving transducer, in order to diagnose the type of contaminant in adulterated milk. According to this methodology, the pattern recognition of a received signal *S*(*t*) can be performed by using three signal parameters: energy (*E*), the average band (*ω_c_*) and RMS bandwidth (*B*). B is also known as the equivalent band, which calculates the standard deviation of the spectrum of a signal. These parameters are defined mathematically as:

(6) $$E\;=\;\frac{1}{2\pi }\overset{N-1}{\underset{n=0}{\sum }}{\left|Y_{n}\right|}^{2}$$

(7) $$\omega_{c}\;=\;\frac{\sum_{n=0}^{N-1}f_{n}{\left|Y_{n}\right|}^{2}}{2\pi E}$$

(8) $$B\;=\;\sqrt{\frac{1}{E}\overset{N-1}{\underset{n=0}{\sum }}{\left(f_{n}-\omega_{c}\right)}^{2}{\left|Y_{n}\right|}^{2}}$$

where *Y_n_* and *f_n_* are, respectively, the Fourier transform and the frequency of the discrete time signal *S*[*n*], and *N* is the length of *S*[*n*].

CT aims to form a data cluster map which can be identifiable from a chromatic point of view [23,24]. Based on the described properties, this article proposes the application of the chromatic technique to classify the type of milk adulteration by industrial contaminants like bicarbonate, urea, and hydrogen peroxide. The idea is to form a color map that relates to the contamination substance. The experimental setup is described in the next section.

## 3. Experimental Setup

**Figure 2:**
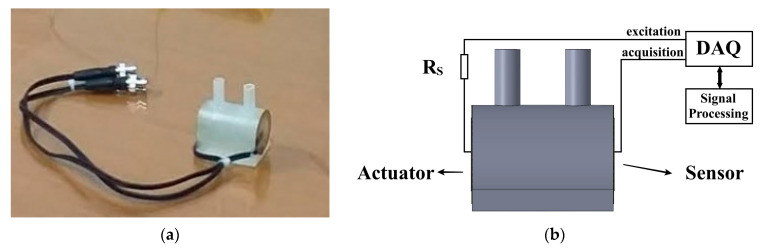
Experimental setup: (**a**) test tube and (**b**) experimental setup diagram. Obs.: Rs is a resistor which limits the current of the DAQ (data acquisition system).

A test tube (34 mm × 60 mm) for milk samples was made on a 3D printer using a polymeric material and two piezoelectric diaphragms were attached to each side of the tube, according to the configuration shown in Figure 2. To apply acoustic testing in milk samples, the transducers were configured as an actuator and sensor, respectively.

**Figure 3:**
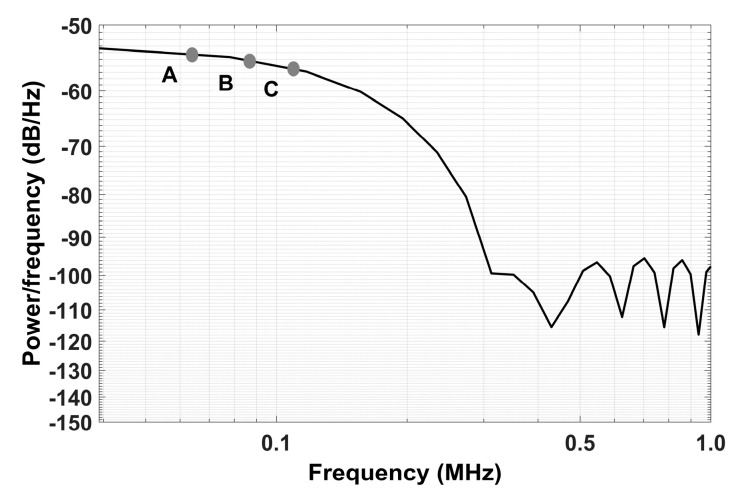
Sensitivity of the piezoelectric transducer in the frequency domain. A, B and C are frequency attenuation of −1 dB, −2 dB and −3 dB, respectively.

Piezoelectric diaphragms used in this work were the 7BB-20-6, manufactured by Murata Electronics^®^ (Kyoto, Japan). They have a circular brass plate whose dimensions are 35 mm × 0.2 mm and a circular piezoelectric ceramic with dimensions 23 mm × 0.2 mm. As demonstrated in experimental and theoretical studies, these piezoelectrics have similar characteristics to conventional PZT ceramics and are known by their low cost and accessibility [25,26,27,28]. To characterize the sensibility of the diaphragms, a pencil lead break test (PLB) was carried out. The PLB is a well-known method, adopted as a standard procedure by the American Society for Testing and Materials (ASTM) [29], and it is used to estimate the impulse response of acoustic transducers. When the pencil lead breaks on the sensor plate, an impulse is produced [29,30,31]. As impulse signals represent a step of infinite frequencies on the frequency domain, this test is commonly used to estimate the sensibility of the acoustic transducer in a range of frequencies. Figure 3 shows power spectral density (PSD) of the average of 10 PLB tests performed in the transducer attached to the test tube. An oscilloscope with the sample rate set 2MHz was used to acquire the signals.

After the test, the frequency analysis was achieved by the percentage of the signal attenuation. In this sense, *PSD* attenuation (*AdB*), is defined in Equation (9), by the initial *PSD* value (*PSD*(0) = 53.47 dB) and the *PSD* for a frequency *f*:

(9) $$AdB\;\;=\;\;20\log\frac{PSD\left(f\right)}{PSD\left(0\right)}$$

Therefore, the percentage of attenuation (%*A*) can be calculated as:

(10) $$\%A\;=\;\left(1-\frac{PSD\left(f\right)}{PSD\left(0\right)}\right)\cdot 100\%\;=\;\left(1-10^{\frac{AdB}{20}}\right)\cdot 100\%$$

According to Figure 3, considering the frequency content attenuation of 1 dB, 2 dB and 3 dB, represented by points “A” (0.06, −54.47) (attenuation of 1 dB in the frequency response of the sensor), “B” (0.08, −55.47) (attenuation of 2 dB in the frequency response of the sensor), and “C” (0.11, −56.47) (attenuation of 2 dB in the frequency response of the sensor). In relation to the first point of the PSD, which is 53.47 dB, it can verified that the sensitivity of the piezoelectric diaphragm dropped 1.87% until 60 kHz, 3.74% until 80 kHz and, 5.6% until 110 kHz. After that, the attenuation increases, and the response of the sensor stop in 300 kHz. 

As the excitation signals were limited until 65 kHz, the attenuation of the system was about 10.87%. Considering further presented results, this sensitivity value presented great potential to detect milk adulteration.

For the test, the generation of acoustic waves by the actuator and its measurement by the sensor were performed using an NI USB-6211 DAQ device (National Instruments, Austin, TX, USA) with a sampling rate of 250 kS/s. The emitter was excited by a chirp signal with an amplitude of 1 V and a frequency range from 0 to 65 kHz with a step of 2 Hz. A resistor (Rs) of 2.2 kΩ ± 1% was connected to limit the current of the DAQ. It can be concluded that the chirp signal has frequencies under the response of the sensor, which is shown in Figure 3.

Raw milk was taken, and then separated into four samples: without contaminant (raw) and adulterated by bicarbonate, urea, and hydrogen peroxide. The percentage of milk contamination was 0.5%, which is a similar value to the frauds found in real applications [1,4,6]. The contamination was assisted by São Paulo State Agribusiness Technology Agency (APTA).

Pure and contaminated milk samples of 50 mL were placed in the tube. For each sample (raw, bicarbonate, urea, and hydrogen peroxide), the test was performed 100 times at 25 °C. We considered one received signal as an average of 10 emitting-receiving steps. Therefore, the waves were propagated 1000 times per milk sample.

The statistical indexes were calculated by comparing an average of 100 signals obtained from pure milk with an average of 100 signals obtained from each contaminated condition. For CT, each cluster related to raw, bicarbonate, urea, and hydrogen peroxide was achieved by the 100 signals extracted.

The instrumentation system proposed in this work takes 0.5 s to emit and receive the signals. Besides, it can be an alternative to portable systems due to its small size (34 mm × 60 mm).

## 4. Results and Discussion

Experimental results are presented in two subsections; Section 4.1 present the results of the statistical indexes, and Section 4.2 discusses the effectiveness of the CT.

### 4.1. Statistical Indexes Results

**Figure 4:**
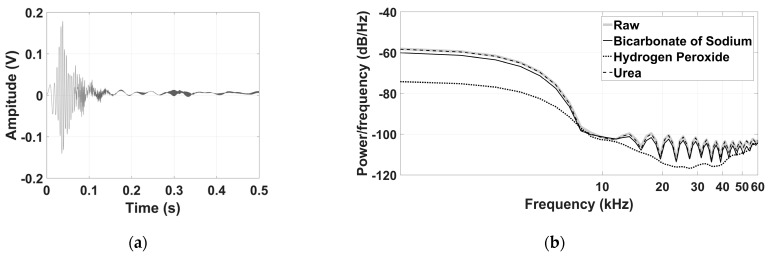
Received signal from non-contaminated milk (**a**). Spectral power density for contaminated and non-contaminated milk (**b**).

Figure 4 shows the received signal in the time domain for pure milk used as reference, and the power spectral density for all cases, contaminated and non-contaminated. The time domain signal decreased 6.67 times regarding the peak of the excitation signal mainly due to the acoustic attenuation caused by bovine milk. Figure 4b shows the power spectral density for all cases. The frequency response curves show that the contamination of bovine milk produced disparities when compared to milk in its natural state. The maximum values of power spectral density were around −58 dB for pure milk and contaminated with urea, while for peroxide and bicarbonate, the maximum values were −60 dB and −74 dB respectively. Although the excitation signal band is 65 kHz, results in Figure 4 show that the most significant frequency range was from 0 to 10 kHz.

**Figure 5:**
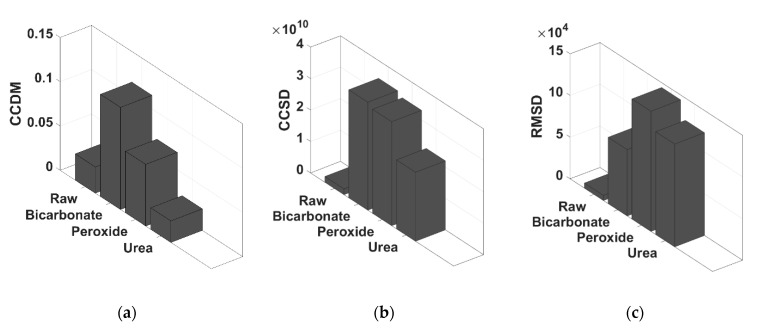
Statistical Indexes for contaminated and non-contaminated milk: (**a**) CCDM, (**b**) CCSD and (**c**) RMSD.

The statistical indexes from data analysis for contaminated and non-contaminated milk are presented in Figure 5.

It is possible to observe that the RMSD and CCSD indexes were effective for detecting adulteration of bovine milk since their values remained around 0 for pure milk samples while showing significative higher values for contaminated milk. RMSD index presented a value of 7 × $10^{4}$ for milk contaminated by sodium bicarbonate, 14 ×$\;10^{4}$ for peroxide, and 12.5 × $10^{4}$ for urea. CCSD index presented a value of 3.3 × $10^{10}$ for bicarbonate contaminant, 3.2 × $10^{10}$ for peroxide, and 2.15 × $10^{10}$ for urea.

Therefore, these indexes can be an alternative to identify the adulteration of milk because these values increase with the presence of adulterants. Furthermore, it can be concluded that CCSD has an average sensitivity of 3.2 × 10^10^ to determine the contamination, against 14 × $10^{4}$ for RMSD, which means that CCSD is 2.28 × $10^{5}$ more sensitivity to distinguish adulteration in relation to RMSD. The CCDM index was not effective, considering that the values presented for pure milk were close to the values with urea. Although CCSD presents higher sensibility, both RMSD and CCSD indexes show effectiveness to identify adulterated milk since the indexes increase with the addition of the contaminant.

### 4.2. Chromatic Technique Applied in Data Separation

**Figure 6:**
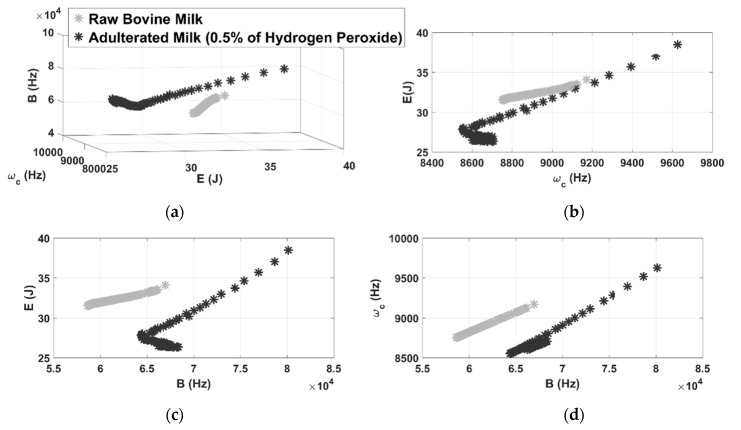
3D representation of color maps for hydrogen peroxide contamination (**a**) and 2D representation of color maps (**b**–**d**).

Figure 6 shows the color map resulted by applying the chromatic technique for adulterated milk with 0.5% of hydrogen peroxide. The 100 tests carried out represents each color map point. As can be seen, the chromatic technique was effective at performing data separation since it clearly produces well-defined different regions, according to the 3D graphic presented in Figure 6a. 

A quantitative comparison can be performed by calculating the shortest distance between clusters in relation to the raw milk inspection. In this case, this value was around 5000. It means that the shortest distance between the clusters is 5000 units. Therefore, the chromatic technique provides significantly greater distance between the clusters formed by non-adulterated milk and adulterated with hydrogen peroxide.

By analyzing the results presented by the 2D clusters (Figure 6b–d), it is observed that the chromatic parameters do not perform the separation of the data in an equivalent weight. As noted in Figure 6b, the regions formed by the pair E × $\omega_{c}$ do not allow the correct diagnosis of adulteration, since the map of pure milk overlaps the map of the contaminant, when E remains between 31 J and 34 J, and $\omega_{c}$ remains between 8750 Hz and 9200 Hz. However, for the other 2D maps (Figure 6c,d) the technique was able to create a distinct region for each set of points formed by the milk measurements.

**Figure 7:**
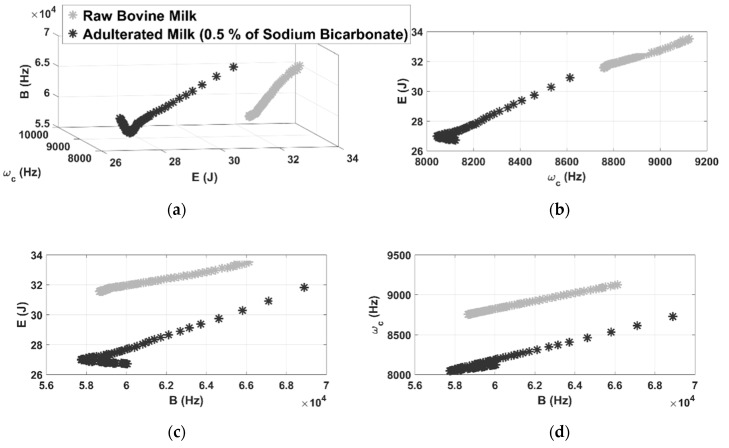
3D representation of color maps for sodium bicarbonate contamination (**a**) and 2D representation of color maps (**b**–**d**).

Figure 7 shows the color map by applying the chromatic technique for adulterated milk with 0.5% sodium bicarbonate. 

As in the previous case, the chromatic technique proved to be effective to identify the adulteration by bicarbonate. In this case, there are two well-defined clusters with a minimum distance (distance of the nearest clusters parameter) of 9000 in relation to the raw milk inspections (Figure 7a). By analyzing the results presented by the 2D clusters, it is observed that the chromatic parameters form distinct separations for all combinations. However, based on the previous results presented by peroxide contamination, 2D clusters are not effective in practical applications, since the cluster formed by$\;E\;\times \;\omega_{c}$ are interconnected (Figure 6b).

**Figure 8:**
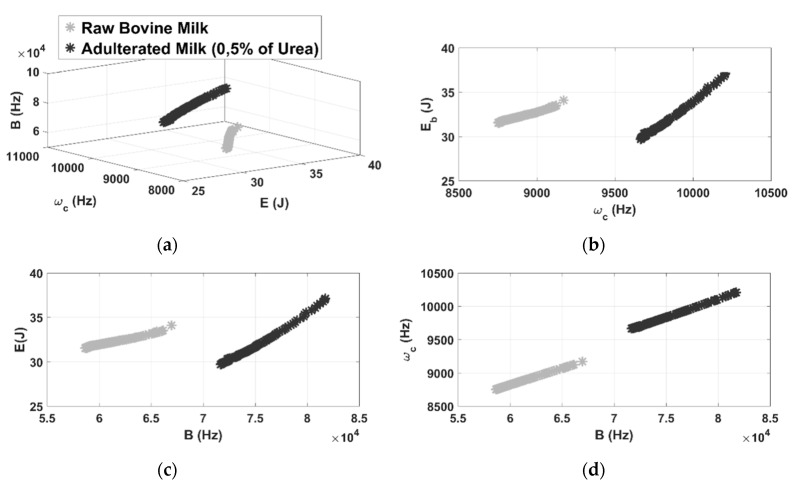
3D representation of color maps for urea contamination (**a**) and 2D representation of color maps (**b**–**d**).

For the clusters formed by the adulterated milk with 0.5% urea, the chromatic technique proved to be effective in diagnosing urea contamination, since there are two well-defined clusters in Figure 8a with the distance of the nearest clusters of 13,000 in relation to the raw milk inspections. By analyzing the results presented by the 2D clusters, it is observed that the chromatic parameters form distinct separations for all combinations, as well as for the case of adulteration by sodium bicarbonate.

**Figure 9:**
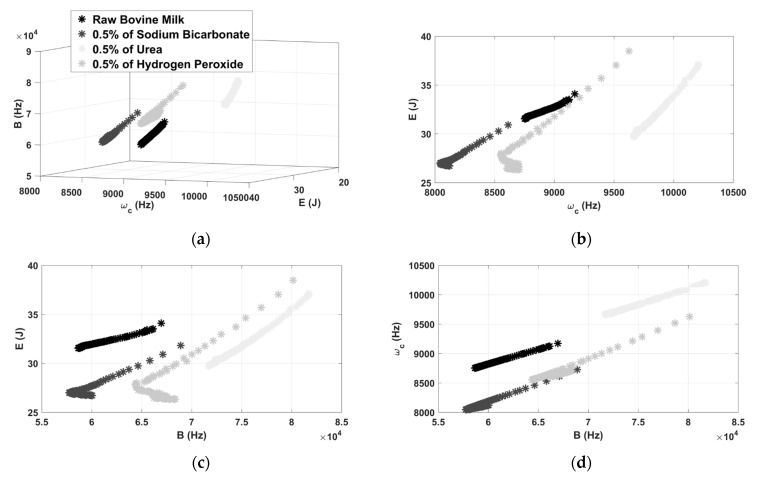
3D representation of color maps for all contaminations (**a**) and 2D representation of color maps (**b**–**d**).

Aiming to verify the effectiveness of CT to classify these types of contaminants, the 3D graphic was shown for raw condition, and contaminated by bicarbonate, urea, and hydrogen peroxide. According to the results shown in Figure 9a, it is possible to observe that the clustering technique is also effective when analyzing all contaminants. Distinct and well-defined clusters were observed for each substance thus making possible the correct identification of the type of contaminant. However, by analyzing the results presented by the 2D clusters ( Figure 9b–d), only the map (*E* × *B*) achieved data separation.

To perform a deeper statistical analysis, Table 1 and Table 2, show, respectively, the standard deviations and the average for all performed tests considering the 100 data points. As explained in the experimental setup section, each point is formed by a response signal taken as an average of 10 emitting-receiving tests.

Considering the standard deviations as a percentage of the average values, the variation of all parameters for all cases was lower than 4%. It means that all measurements produced ranges lower than 4% in relation to the average of 100 tests performed. Only the Energy (*E*) extracted in hydrogen peroxide test presented a deviation of 5% of the average of the test.

**Figure 10:**
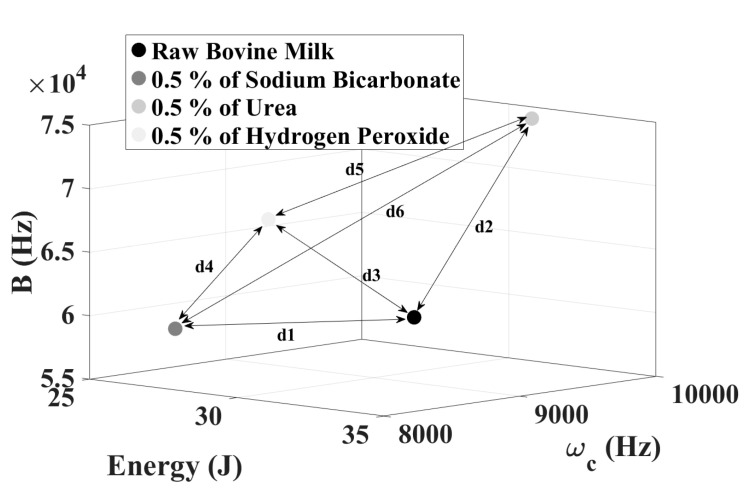
Average coordinates points and their distance of each contamination.

Besides to previous selectivity assessment made by the shortest distance metric, the average point of each cluster was calculated and presented as 3D-graphic according to Figure 10. The coordinates ($\omega_{c}$, *E*, *B*) for raw milk and adulterated by bicarbonate, urea, and hydrogen peroxide were, respectively: (8850.2, 32.1, 60,597.1), (8133.1, 27.3, 59,427.2), (9815.1, 31.63, 74,605.6) and (8708.9, 27.8, 67,286.4). To achieve a quantitative analysis, the parameter *dn* (1 ≤ *dn* ≤6), shown in Figure 10, was defined as the Euclidian distance between the average point of all clusters. It can be observed that the proposed methodology is a promising milk adulteration detection tool. The distance between raw milk and the sodium bicarbonate coordinates (*d*1) was 1372.20. For urea contamination (*d*2) was 14,041.69. The selectivity for hydrogen peroxide (*d*3), calculated by the distance between the points of raw milk condition and adulterated (*d*3), dropped to 6690.79. 

Based on these results it can be concluded that the selectivity of the method is higher for sodium bicarbonate and urea, compared to adulterations carried out by hydrogen peroxide.

Concerning to the distance between the points related to only contaminated conditions, the distance between sodium bicarbonate and peroxide (*d*4) was 7880.27. For hydrogen peroxide and urea (*d*5), 7402.32. Major selectivity was observed between the contamination performed by sodium bicarbonate and urea (*d*6), which was 15,271.31. 

The methodologies proposed by this work make it possible to assess the condition of the bovine milk by analyzing statistical indexes such as CCSD and RMSD. Such method can also be applied in the industrial food system to detect disparities in the pattern of production. Besides, the types of contaminations considered in this work can be identified by chromatic technique analysis, which produced well-defined regions related to milk condition.

## 5. Conclusions

This article proposes a new method for diagnosing bovine milk adulteration based on the application of the acoustic method, using low-cost piezoelectric sensors associated with digital signal processing techniques. The results indicated that the parameters used were effective in diagnosing and differentiating pure milk from that contaminated with low concentrations of hydrogen peroxide, sodium bicarbonate and urea, demonstrating that this technique may have significant applicability as a quality control tool for industrial applications. The CCSD and RMSD indexes prove to be effective to distinguish the raw and adulterated milk by comparing the patterns of the response signals. Regarding classification, the CT formed separation maps which permitted the differentiation of types of the contaminants. Therefore, the proposed technique constitutes an effective approach to assess the milk condition and classify its contaminants using portable and fast systems. Further analysis can study the influence of temperature variations, noise, and humidity in the proposed method. The capability of the system to sense the concentration evolution needs to be analyzed. Also, the minimum detectable contamination and more types of adulterants (chemical or biological) should be considered to broaden this promising technique. Besides these limitations, the proposed instrumentation system needs to be tested in other kinds of milks.

## Figures and Tables

**Table 1:** Standard deviation of the tests.

Milk Samples				
CT	Raw	Sodium Bicarbonate	Urea	Hydrogen Peroxide
***E***	0.6	0.9	1.2	1.34
$\omega_{c}$	116.9	118.9	157.6	190.8
B	2336.9	1885.6	2935.4	2477.5

**Table 2:** Average of the tests.

Milk Samples				
CT	Raw	Sodium Bicarbonate	Urea	Hydrogen Peroxide
***E***	32.1	27.3	31.63	27.8
$\omega_{c}$	8850.2	8133.1	9815.1	8708.9
B	60,597.1	59,427.2	74,605.6	67,286.4
